# Neuroprotective effects of bilobalide on cerebral ischemia and reperfusion injury are associated with inhibition of pro-inflammatory mediator production and down-regulation of JNK1/2 and p38 MAPK activation

**DOI:** 10.1186/s12974-014-0167-6

**Published:** 2014-09-26

**Authors:** Mingjin Jiang, Jing Li, Qiuxian Peng, Yi Liu, Wei Liu, Chaohua Luo, Ju Peng, Junkui Li, Ken Kin Lam Yung, Zhixian Mo

**Affiliations:** School of Traditional Chinese Medicine, Southern Medical University, Guangzhou, 510515 China; Department of Biology, Hong Kong Baptist University, Kowloon Tong, Hong Kong

**Keywords:** bilobalide, cerebral ischemia and reperfusion, p-ERK1/2, p-JNK1/2, p-p38 MAPK, pro-inflammatory mediators

## Abstract

**Background:**

Mitogen-activated protein kinase (MAPK) signaling pathways are implicated in inflammatory and apoptotic processes of cerebral ischemia and reperfusion (I/R) injury. Hence, MAPK pathways represent a promising therapeutic target. Exploring the full potential of inhibitors of MAPK pathways is a useful therapeutic strategy for ischemic stroke. Bilobalide, a predominant sesquiterpene trilactone constituent of *Ginkgo biloba* leaves, has been shown to exert powerful neuroprotective properties, which are closely related to both anti-inflammatory and anti-apoptotic pathways. We investigated the neuroprotective roles of bilobalide in the models of middle cerebral artery occlusion and reperfusion (MCAO/R) and oxygen-glucose deprivation and reoxygenation (OGD/R) of cerebral I/R injury. Moreover, we attempted to confirm the hypothesis that its protection effect is via modulation of pro-inflammatory mediators and MAPK pathways.

**Methods:**

Male Sprague-Dawley rats were subjected to MCAO for 2 h followed by reperfusion for 24 h. Bilobalide was administered intraperitoneally 60 min before induction of middle cerebral artery occlusion (MCAO). After reperfusion, neurological deficit scores, infarct volume, infarct weight, and brain edema were assessed. Ischemic penumbrae of the cerebral cortex were harvested to determine superoxide dismutase (SOD), malondialdehyde (MDA), nitric oxide, TNF-α, interleukin 1β (IL-1β), p-ERK1/2, p-JNK1/2, and p-p38 MAPK concentration. Similarly, the influence of bilobalide on the expression of nitric oxide, TNF-α, IL-1β, p-ERK1/2, p-JNK1/2, and p-p38 MAPK was also observed in an OGD/R *in vitro* model of I/R injury.

**Results:**

Pretreatment with bilobalide (5, 10 mg/kg) significantly decreased neurological deficit scores, infarct volume, infarct weight, brain edema, and concentrations of MDA, nitric oxide, TNF-α, IL-1β, and increased SOD activity. Furthermore, bilobalide (5, 10 mg/kg) pretreatment significantly down-regulated both p-JNK1/2 and p-p38 MAPK expression, whereas they had no effect on p-ERK1/2 expression in the ischemic penumbra. Supporting these observations *in vivo*, pretreatment with bilobalide (50, 100 μM) significantly down-regulated nitric oxide, TNF-α, IL-1β, p-JNK1/2, and p-p38 MAPK expression, but did not change p-ERK1/2 expression in rat cortical neurons after OGD/R injury.

**Conclusions:**

These data indicate that the neuroprotective effects of bilobalide on cerebral I/R injury are associated with its inhibition of pro-inflammatory mediator production and down-regulation of JNK1/2 and p38 MAPK activation.

## Background

Ischemic stroke, also known as cerebral infarction, is a common and life-threatening cerebrovascular disease with substantial morbidity and mortality worldwide [1,2]. Ischemic stroke due to sudden occlusion of a blood vessel by a thrombus or embolism accounts for 87% of all stroke cases [1]. Rapid reperfusion is critical in the treatment of unexpected cerebral ischemic incidents. However, perhaps surprisingly, the occurrence of post-perfusion lesions is usually associated with exacerbation of brain injury and a profound inflammatory response [3,4]. Although the mechanisms of cerebral ischemia and reperfusion (I/R) injury are complex and involve the interaction of numerous pathophysiological processes, there is accumulating evidence that inflammation and apoptosis are involved [3-6].

Mitogen-activated protein kinases (MAPKs) are activated after focal cerebral I/R and play crucial roles in regulating neuronal survival or damage [7-10]. The activated MAPKs mainly function as mediators of cellular stress by phosphorylating intracellular enzymes, transcription factors, and cytosolic proteins involved in cell survival, inflammatory mediators production, and apoptosis [11,12]. Extracellular signal-regulated kinases (ERKs), c-Jun NH_2_-terminal kinases (JNKs), and p38 kinases are the best-known MAPK systems. In contrast with ERK, which is part of the survival route, the presence of JNK and p38 MAPK could have an impact on cell injury [11]. There is a growing body of evidence to show that p38 MAPK is activated in neurons, astrocytes, and microglia after various types of ischemia [13-15], and its prolonged activation is associated with neuronal apoptosis and the production of pro-inflammatory cytokines, such as tumor necrosis factor α (TNF-α) and interleukin 1β (IL-1β), which are favored by acting as perpetrators in the central nervous system injury as well as conversely activating the p38 MAPK pathway [16,17]. Furthermore, inhibition of p38 MAPK activation has been demonstrated to provide protection in a variety of *in vitro* and *in vivo* models of brain injury [18-22]. Activation of JNK is induced in the brain after focal ischemia [7-10]. Cumulative evidence from experiments using JNK inhibitors or JNK knockout mice reveals a pivotal role of JNK in neuronal apoptosis and a benefit of the inhibitors in focal stroke models [23-26]. Phosphorylation of ERK occurs at different time intervals after I/R injury [8,9]. However, whether the activation of ERK is associated with neuronal protection or damage in ischemic brain remains to be determined unequivocally [27,28]. Taken together, these results indicate that the activation of MAPK families is involved in the process of ischemia-induced neuronal injury. Thus, the studies of MAPK activation in ischemic brain may provide fertile ground for the discovery of novel therapeutic agents for stroke patients.

**Figure 1:**
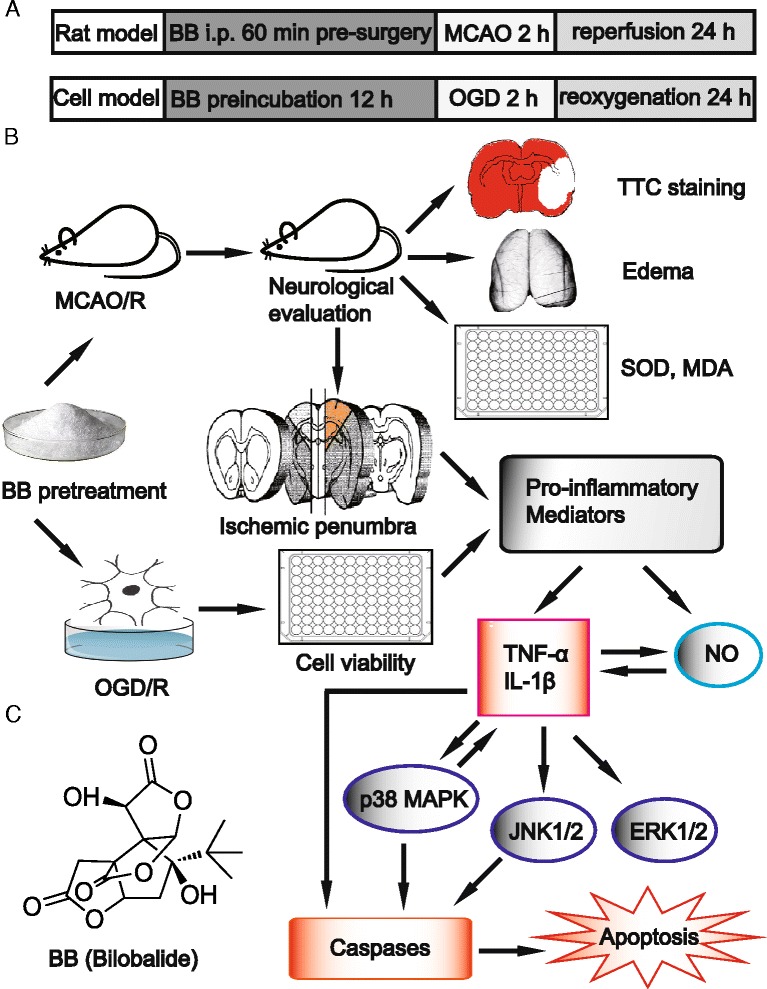
**Chemical structure of bilobalide and experimental protocol. (A)** Rat MCAO/R and *in vitro* OGD/R model of cerebral ischemia and reperfusion injury. Bilobalide (2.5, 5, and 10 mg/kg) was administered via a single intraperitoneal injection 60 min prior to surgery in the rat model. In the cell model, cortical neurons were previously cultured in bilobalide (50, 100 μM) for 12 h by dissolving bilobalide in serum-free DMEM. **(B)** Experimental protocol; neuroprotective effects of bilobalide on cerebral ischemia and reperfusion injury are associated with pro-inflammatory mediator production and MAPK signaling pathway. **(C)** Chemical structure of bilobalide. BB, bilobalide; MCAO/R, middle cerebral artery occlusion and reperfusion; MDA, malondialdehyde; OGD/R, oxygen-glucose deprivation and reoxygenation; SOD, superoxide dismutase; TTC, 2,3,5-triphenyltetrazolium chloride.

Bilobalide (Figure 1C) is a predominant sesquiterpene trilactone constituent that accounts for 2.9% of the standardized *Ginkgo biloba* extract EGb 761, which has been widely used to treat a variety of neurological disorders involving cerebral ischemia and neurodegeneration [29,30]. Substantial experimental evidence indicates that bilobalide possesses many beneficial effects, such as neuroprotective, anti-inflammatory, anti-apoptotic, and anticonvulsant effects in various models [31-34]. Bilobalide has recently attracted considerable interest, owing to its potent effects on the central nervous system, such as acting as a noncompetitive inhibitor of γ-aminobutyric acid, glycine, and 5-HT_3_ receptors [35-38]. Bilobalide has been demonstrated to reduce infarct areas and edema formation after focal cerebral ischemia in rodents [31,39], antagonize neuronal damage [40], and accelerate the regeneration of rat motor neurons in cell culture [41]. Several recent reports have shown that bilobalide can attenuate neuronal inflammation and apoptosis in the frontal cortex and hippocampus CA1 in a rat model of Alzheimer’s disease [42], reduce ischemia-induced glutamate release in both core and penumbral regions [43], significantly enhance hippocampal neuronal proliferation and synaptogenesis, and protect against amyloid-β oligomer-induced synaptic loss by modulating phosphorylation of the cyclic-AMP response element binding protein [44]. In addition, bilobalide prevents apoptosis through activation of the PI3K/Akt pathway in SH-SY5Y cells [45]. Together, these studies clearly show that the neuroprotective effects of bilobalide are closely related to both anti-inflammatory and anti-apoptotic pathways, although its specific mechanisms are not well understood.

In this study, we hypothesized that MAPK pathways could be a therapeutic target of bilobalide in conditions of cerebral I/R injury. We investigated, therefore, the effects of bilobalide on the production of pro-inflammatory mediators and the expression of p-ERK1/2, p-JNK1/2, and p-p38 MAPK in the ischemic penumbra of the cerebral cortex after middle cerebral artery occlusion and reperfusion (MCAO/R) injury in rats as well as in rat cortical neurons after oxygen-glucose deprivation and reoxygenation (OGD/R, Figure 1A,B).

## Methods

### Experimental animals and middle cerebral artery occlusion and reperfusion (MCAO/R) model

Adult male Sprague-Dawley rats, weighing 280 to 320 g, were provided by the Laboratory Animal Center of Southern Medical University (Guangzhou, China) and housed under diurnal lighting conditions (12-hour light/dark cycle). All experimental protocols and animal handling procedures were performed in accordance with the National Institutes of Health USA Guide for the Care and Use of Laboratory Animals, and were approved by the Experimental Animal Ethics Committee of Southern Medical University.

Rats were allowed free access to food and water but were fasted 12 h before surgery. All animals were anesthetized by intraperitoneal injection of sodium pentobarbital (45 mg/kg). The MCAO/R model was performed as described previously, with minor modifications [46]. Briefly, the right common carotid artery, internal carotid artery, and external carotid artery were exposed through a ventral midline neck incision. The internal carotid artery was then isolated and coagulated, and the proximal common carotid artery was ligated. A 4-0 monofilament nylon suture (Beijing Sunbio Biotech Co. Ltd., Beijing, China) with a rounded tip was inserted into the internal carotid artery from the common carotid artery through the external carotid artery stump and gently advanced 18 to 20 mm to occlude the middle cerebral artery. After 2 h of MCAO, the suture was removed to restore blood flow (reperfusion). The rats were placed into cages to recover after incision closure, with free access to food and water. Sham-operated rats underwent identical surgery except that the suture was not inserted. Core body temperature was maintained at 37.0 ± 0.5°C using a heating pad and heating lamp during the whole procedure.

Rats were divided randomly into six groups: sham group, MCAO/R group, and treatment groups, which were pretreated with bilobalide (with purity ≥ 96.0%, Sigma-Aldrich, St. Louis, MO, USA) at doses of 2.5, 5, and 10 mg/kg and nimodipine (Bayer, Leverkusen, Germany) at a dose of 4 mg/kg (positive control), respectively. Brain levels of bilobalide increase for the dose range 1 to 10 mg/kg, but they decrease for higher doses (20 and 40 mg/kg) [47]. Referring to a previous study [48] and our pre-test, we determined the doses (2.5, 5, and 10 mg/kg) of bilobalide. Nimodipine is a cerebroselective calcium channel blocker, which readily crosses the blood-brain barrier and has been shown to exert neuroprotective effects in cerebral I/R injury [49,50]. The neuroprotective mechanism of nimodipine may be related to both anti-inflammatory and anti-apoptotic pathways. Nimodipine may protect the penumbral region from becoming necrotic. Therefore, we used nimodipine as a positive control drug. It has been well proved that nimodipine at 4 mg/kg is a high and effective dose in cerebral ischemia and reperfusion injury, and the dose is very close to the effective dose of bilobalide [50,51]. Bilobalide (2.5, 5, and 10 mg/kg, in saline containing 10% dimethyl sulfoxide (DMSO)) and nimodipine (4 mg/kg) were administered via a single intraperitoneal injection 60 min prior to surgery.

### Evaluation of neurological deficit

Neurological deficit was evaluated 24 h after reperfusion by an investigator who was unaware of animal grouping. A five-point scale of neurologic deficit scores was used to evaluate neurological behavior, referring to the methods of Longa *et al*. and Bederson *et al*. [52,53] (Table 1).

**Table 1:** **Neurological deficit scores on a five-point scale in rats**

**Grade**	**Points**	**Symptoms of neurological behavior**
Normal (no neurological deficit)	0	Rats behave normally, can fully outstretch forelimbs toward the ground when lifting tails and dangling.
Mild neurological deficit	1	Rats cannot fully stretch their left forelimbs when lifting tails and dangling.
Moderate neurological deficit	2	Rats have decreased resistance to lateral push and mild neurological behavior.
Severe neurological deficit	3	Rats turn around into a circle, and have mild and moderate neurological behavior.
	4	Rats do not walk spontaneously and have a depressed level of consciousness.

### Quantification of infarct volume, infarct weight, and brain water content

After neurological evaluation, rats were decapitated and the brains were rapidly removed and mildly frozen to keep the morphology intact during slicing. Infarct volume was measured as described previously [54]. In brief, brains (*n* = 6 for each group) were cut into 2-mm-thick coronal sections in a brain matrix and stained with 2% (w/v) 2,3,5-triphenyltetrazolium chloride (TTC) (Sigma-Aldrich, St. Louis, MO, USA) for 30 min at 37°C followed by overnight immersion in 4% (w/v) paraformaldehyde. The infarct tissue area remained unstained (white), whereas normal tissue was stained red. The infarct areas on each slice were demarcated and analyzed by Image J software (National Institutes of Health, Bethesda, MD, USA). To compensate for the effect of brain edema, the corrected infarct volume was calculated as follows [46]:

$$ \begin{array}{l}\mathrm{Percentage}\kern0.5em \mathrm{of}\kern0.5em \mathrm{corrected}\kern0.5em \operatorname{infarct}\kern0.5em \mathrm{volume}\kern0.5em =\kern0.5em \left\{\right[\mathrm{Contralateral}\kern0.5em \mathrm{hemisphere}\kern0.5em \mathrm{area}\kern0.5em -\kern0.5em \Big(\mathrm{Ipsilateral}\\ {}\mathrm{hemisphere}\kern0.5em \mathrm{area}\kern0.5em -\kern0.5em \mathrm{Measured}\kern0.5em \operatorname{infarct}\kern0.5em \mathrm{area}\left)\right]\;/\;\mathrm{Contralateral}\kern0.5em \mathrm{hemisphere}\kern0.5em \mathrm{area}\Big\}\kern0.5em \times \kern0.5em 100\%\end{array} $$

To evaluate the infarction areas further, the infarct tissue areas were separated from normal tissue using a blade. Similarly, the corrected infarct weight was expressed as follows:

$$ \begin{array}{l}\mathrm{Percentage}\kern0.5em \mathrm{of}\kern0.5em \mathrm{corrected}\kern0.5em \mathrm{infarct}\kern0.5em \mathrm{weight}\kern0.5em =\kern0.5em \left\{\right[\mathrm{Contralateral}\kern0.5em \mathrm{hemisphere}\kern0.5em \mathrm{weight}-\Big(\mathrm{Ipsilateral}\\ {}\mathrm{hemisphere}\kern0.5em \mathrm{weight}-\mathrm{Measured}\kern0.2em \mathrm{infarct}\kern0.5em \mathrm{weight}\left)\right]\kern0.2em /\kern0.2em \mathrm{Contralateral}\kern0.5em \mathrm{hemisphere}\kern0.5em \mathrm{weight}\Big\}\kern0.5em \times \kern0.5em 100\%\end{array} $$

After brain extraction, animals observed to have experienced a subarachnoid hemorrhage were excluded from the study.

Brain water content was determined by the wet-dry method in another subgroup (*n* = 6 for each group) after neurological assessment [39]. The ipsilateral and contralateral hemispheres were weighed with an electronic scale (wet weight) and dried overnight at 105°C in a desiccating oven. The dried brain hemispheres were weighed again (dry weight), and the total brain water content was calculated according to:

$$ \left[\left(\mathrm{Wet}\kern0.5em \mathrm{weight}\kern0.5em -\kern0.5em \mathrm{Dry}\;\mathrm{weight}\right)\;/\;\mathrm{Wet}\;\mathrm{weight}\right]\kern0.5em \times \kern0.5em 100\% $$

### Ischemic penumbra dissection

After neurological assessment, rats were decapitated and the brains were rapidly removed. The ischemic penumbra of the cerebral cortex was determined according to the methods as previously described [55]. Briefly, the brain was sectioned into three slices, starting 3 mm from the anterior tip of the frontal lobe in the coronal plane. The slices were 3, 4, and 3 mm thick from front to back, respectively. The middle slice was cut longitudinally in the ischemic hemisphere 2 mm from the midline, and then a transverse diagonal cut was made at the 2 o’clock position to separate the core from the penumbra (Figure 1B).

### Measurements of the levels of superoxide dismutase and malondialdehyde

The ischemic penumbra was dissected out and homogenized with cold normal saline, centrifuged at 12,500 *g* for 10 min at 4°C, and the supernatant was collected for assays. The antioxidant status of the brain was assessed by the activity of superoxide dismutase (SOD). The lipid peroxidation was determined by the concentration of malondialdehyde (MDA), an end product of lipid peroxidation, according to the kit instructions (Nanjing Jiancheng Bioengineering Institute, Nanjing, China). The activity of SOD was evaluated by the xanthine oxidase method. Briefly, 1 ml of Reagent 1 was added to measure and control tubes, respectively. Then, 0.2 ml of samples were added to measure tubes and 0.2 ml distilled water was added to control tubes. Next, 0.1 ml of Reagent 2, 3, 4 were added to measure and control tubes, respectively. The solution was mixed and incubated at 37°C for 40 min in a water bath and 2 ml chromogenic agents were added per tube. After mixing, the solution was placed at room temperature for 10 min. The absorbance was determined at 550 nm with a spectrophotometer (Bio-Rad Laboratories, Hercules, CA, USA). The SOD activity was calculated as follows:

$$ \begin{array}{l}\mathrm{SOD}\kern0.5em \left(\mathrm{units}/\mathrm{mg}\kern0.5em \mathrm{protein}\right)\kern0.5em =\kern0.5em \left\{\right[\left(\mathrm{Control}\kern0.5em \mathrm{optical}\kern0.5em \mathrm{density}-\mathrm{Measured}\;\mathrm{optical}\;\mathrm{density}\right)\kern0.2em /\kern0.2em \mathrm{Control}\\ {}\mathrm{optical}\kern0.5em \mathrm{density}\;\mathrm{value}\Big]\kern0.5em /\kern0.2em 50\%\times \kern0.5em \left(\mathrm{Reaction}\;\mathrm{solution}\;\mathrm{volume}\;/\;\mathrm{Sample}\;\mathrm{volume}\right)\;/\;\mathrm{Protein}\\ {}\mathrm{concentration}\;\mathrm{of}\ \mathrm{the}\ \mathrm{sample}\Big\}\end{array} $$

The MDA content was assayed using a thiobarbituric acid method. Briefly, the same volumes (0.2 ml) of anhydrous ethanol, standard solution, measure solution, and measure solution were added to the blank, standard, measure, and control tubes, respectively. Then 0.2 ml Reagent 1 and 3 ml Reagent 2 were added per tube, 1 ml Reagent 3 was added to the blank, standard, and measured tubes, and 1 ml 50% glacial acetic acid was added to the control tube. The solution was mixed and incubated at 95°C for 40 min in a water bath. After cooling, the solution was centrifuged at 4,000 *g* for 10 min. The absorbance of the supernatant was determined at 532 nm using a spectrophotometer (Bio-Rad Laboratories, Hercules, CA, USA). The MDA concentration was determined as follows:

$$ \begin{array}{l}\mathrm{M}\mathrm{D}\mathrm{A}\;\left(\mathrm{nmol}/\mathrm{mg}\;\mathrm{protein}\right)\kern0.5em =\left[\mathrm{Measured}\;\mathrm{optical}\ \mathrm{denstity}-\mathrm{Control}\;\mathrm{optical}\;\mathrm{density}\right)\;/\;\Big(\mathrm{Reference}\\ {}\mathrm{standard}\;\mathrm{optical}\;\mathrm{density}-\mathrm{Blank}\;\mathrm{optical}\;\mathrm{density}\left)\right]\times \mathrm{Reference}\;\mathrm{standard}\;\mathrm{concentration}\kern0.2em /\\ {}\mathrm{Protein}\;\mathrm{concentration}\;\mathrm{of}\;\mathrm{the}\ \mathrm{sample}\end{array} $$

### Primary cortical neuron culture and oxygen-glucose deprivation and reoxygenation (OGD/R)

Primary cultures of cortical neurons were obtained from fetal rats at 17 days of gestation. The procedures have been described previously [56]. Briefly, cerebral cortices were isolated and digested in 0.25% trypsin. The resulting cell suspension was plated onto poly-L-lysine pre-coated wells and cultivated in DMEM with 10% FCS and 10% fatal horse serum in a humidified atmosphere of 95% air and 5% CO_2_ at 37°C. Cytosine-D-arabinofuranoside (10 μM) was added to the cultures 36 h after plating, to avoid proliferation of non-neuronal elements, and was kept for 2 days before medium replacement. Only mature (10 to 12 days *in vitro*) cultures were used for experiments. The purity of the neuronal cultures was confirmed by microtubule associated proteins-2 (MAP-2) staining [57]. Briefly, cultures (10 to 12 days) were fixed for 30 min in 4% paraformaldehyde/PBS, and then washed three times with PBS buffer. Cells were permeabilized with 0.01% Triton X-100. After washing, cultures were blocked for 30 min with 3% horse serum, and then incubated for 2 h at room temperature with monoclonal mitogen-activated protein (MAP)-2 antibodies (1:500 dilution, Sigma-Aldrich, St. Louis, MO, USA). After washing, FITC-conjugated anti-rabbit secondary antibodies (1:100 dilution, Proteintech Group, Chicago, IL, USA) were incubated for 45 min. Cells were observed in an inverted fluorescence microscope. Digital images of marked fields stained for MAP-2 were saved and analyzed. Over 95% of the cells in the cultures were neurons, as determined by immunostaining of the neuron-specific marker MAP-2.

Previous studies have indicated that bilobalide (25 to 100 μM) can dose-dependently protect neurons against oxidative stress, and that incubation of cells with bilobalide alone at 100 μM does not affect cell viability [40]. In a pre-test, we found that pretreatment of primary cultured neurons with bilobalide at 50 and 100 μM significantly increased cell viability. Pretreatment with bilobalide at 25 μM showed an increase in cell viability. However, the change did not prove to be significant. A higher concentration (>100 μM) may affect cell viability. Therefore, we used bilobalide at concentrations of 50 and 100 μM in the final experiment.

Oxygen-glucose deprivation and reoxygenation was performed as reported previously with minor modifications [58]. Cortical neurons were previously cultured in bilobalide (50, 100 μM) for 12 h by dissolving bilobalide in serum-free DMEM (bilobalide was dissolved in DMSO and added to the medium, and DMSO at the final concentrations used was less than 0.1%). The cultures were then incubated with a glucose-free Earle’s balanced salt solution (BSS) (containing a deoxygenated reagent, 0.5 mmol/l sodium dithionite) and immediately transferred to a humidified anaerobic chamber for 2 h (Reming Bioinstrument, Redfield, NY, USA) perfused with 95% N_2_ and 5% CO_2_ at 37°C. Reoxygenation was induced by quickly replacing the deoxygenated and glucose-free BSS with the pre-OGD culture medium and returning the cells to normoxic conditions. Control sister culture plates were exposed to oxygenated BSS containing 5.5 mM glucose in normoxic conditions during the same period as the OGD cultures.

### Assessment of cell viability

The cell viability was assessed by 3-(4,5-dimethylthiazol-2-yl)-2,5-diphenyl tetrazolium bromide (MTT) assay. At 24 h after reoxygenation, MTT solution (5 mg/ml; 10 μl/well) was added, and cells were incubated for an additional 4 h at 37°C. Subsequently, DMSO (200 μl/well) was added to each well for 10 min, to dissolve the formazan crystals. The absorbance was measured at 490 nm with an ELISA plate reader (Bio-Rad Laboratories, Hercules, CA, USA). Cell viability was expressed as the percentage of viable cells in OGD/R with bilobalide pretreatment plates compared with control normoxic plates, as determined by MTT reduction. Each experiment was repeated in triplicate using three independent cultures.

To increase the reliability of the MTT results obtained, lactate dehydrogenase (LDH) release was determined using an LDH assay kit (Nanjing Jiancheng Bioengineering Institute, Nanjing, China) according to the manufacturer’s instructions. At 24 h after reoxygenation, the culture media were harvested and lysed with 0.1% Triton X-100 for 30 min at 37°C to release the intracellular LDH. The absorbance was measured at 490 nm with a spectrophotometer (Bio-Rad Laboratories, Hercules, CA, USA). The LDH release was expressed as a percentage of LDH values of total neurons.

### Measurements of nitric oxide, TNF-α, and IL-1β concentrations

Nitric oxide, TNF-α, and IL-1β concentrations were measured in the ischemic penumbra of the cerebral cortex and cortical neurons. Briefly, the penumbral sections were collected and homogenized through sonication: the samples were quickly frozen and stored at −20°C until assayed for TNF-α and IL-1β using a commercially available ELISA kit (Toray Fujibionics, Tokyo, Japan). Similarly, the secretion of TNF-α and IL-1β into the culture supernatant was measured using the ELISA kits. The nitric oxide concentration was expressed as nitrites and nitrates (nitric oxide metabolites), which were detected using a nitric oxide nitrate reductase assay kit (Nanjing Jiancheng Bioengineering Institute, Nanjing, China) according to the manufacturer’s protocol. The nitric oxide concentration was calculated as follows in tissue samples:

$$ \begin{array}{l}\mathrm{Nitric}\kern0.5em \mathrm{oxide}\kern0.5em \left(\upmu \mathrm{mol}/\mathrm{g}\;\mathrm{protein}\right)\kern0.5em =\kern0.5em \Big[\left(\mathrm{Measured}\kern0.5em \mathrm{optical}\kern0.2em \mathrm{density}-\mathrm{Control}\kern0.2em \mathrm{optical}\kern0.2em \mathrm{density}\right)\kern0.2em /\kern0.2em \\ {}\left(\mathrm{Reference}\;\mathrm{standard}\;\mathrm{optical}\;\mathrm{density}\kern0.5em -\kern0.5em \mathrm{Control}\;\mathrm{optical}\;\mathrm{density}\right)\Big]\times \mathrm{Reference}\;\mathrm{standard}\\ {}\mathrm{concentration}\;/\;\mathrm{Protein}\;\mathrm{concentration}\;\mathrm{of}\ \mathrm{the}\ \mathrm{sample}\end{array} $$

and as follows in cell samples:

$$ \begin{array}{l}\mathrm{Nitric}\kern0.2em \mathrm{oxide}\ \left(\upmu \mathrm{mol}/\mathrm{l}\right)=\Big[\left(\mathrm{Measured}\ \mathrm{optical}\kern0.2em \mathrm{density}-\mathrm{Control}\kern0.2em \mathrm{optical}\ \mathrm{density}\right)\ /\ \mathrm{Reference}\\ {}\mathrm{standard}\ \mathrm{optical}\ \mathrm{density}-\mathrm{Control}\kern0.2em \mathrm{optical}\ \mathrm{density}\left)\right]\times \mathrm{Reference}\kern0.2em \mathrm{standard}\kern0.2em \mathrm{concentartion}\times \\ {}\mathrm{Dilution}\ \mathrm{multiple}\ \mathrm{of}\ \mathrm{the}\ \mathrm{sample}\end{array} $$

### Isolation of proteins and Western blot analysis

The proteins in the ischemic penumbra of cerebral cortex and cortical neurons were isolated, as previously described [59]. Briefly, the samples were homogenized in lysis buffer and centrifuged at 20,000 *g* for 15 min at 4°C. The supernatants were collected and employed for protein determination using the Beyotime Protein Assay Kit (Beyotime Institute of Biotechnology, Nanjing, China). As previously described in detail, [59] protein samples were denatured in reducing buffer and separated on 10% sodium dodecyl sulfate-polyacrylamide gel electrophoresis gels (20 to 50 μg/lane) and then transferred to a polyvinylidene fluoride membrane (Millipore Corporation, Billerica, USA). The membrane was blocked with 5% nonfat dry milk in Tris-buffered saline containing 0.05% Tween-20 (TBST) buffer and then incubated with primary antibodies for p-ERK1/2 (p: phosphorylated, active form), p-JNK1/2, p-p38 MAPK (1:500 dilution), total ERK1/2, total JNK1/2, and total p38 MAPK (1:1000 dilution, Santa Cruz Biotechnology, Santa Cruz, CA, USA) overnight at 4°C. The following day, the membranes were washed three times with TBST buffer and incubated with secondary antibodies coupled to horseradish peroxidase (1:1000 dilution, Santa Cruz Biotechnology) for 2 h at room temperature. To prove equal loading, the blots were analyzed for β-actin (housekeeping gene) expression using an anti-β-actin antibody (1:500 dilution, Santa Cruz Biotechnology). After washing, the membranes were analyzed by the enhanced chemiluminescence system according to the manufacturer’s protocol (Millipore Corporation, Billerica, USA). Protein signals were quantified by scanning densitometry using Quantity One Software (Bio-Rad Laboratories, Hercules, CA, USA). The levels of total ERK1/2, total JNK1/2, and total p38 MAPK were expressed as relative integrated intensity normalized versus β-actin. The p-ERK1/2, p-JNK1/2, and p-p38 MAPK signals were shown as the ratio of the integrated intensity of the phosphorylated versus the unphosphorylated form.

### Statistical analysis

Values are expressed as mean ± standard error of the mean. All data were analyzed using one-way analysis of variance (ANOVA), followed by the least significant difference post hoc test (two-tailed). All statistical analyses were performed using SPSS software (version 13.0). *P* < 0.05 was considered to be statistically significant. Neurological deficit scores were expressed as median (range). The neurological deficit scores among different groups were compared using the Kruskal-Wallis test. When the Kruskal-Wallis test showed significant difference, the Mann-Whitney *U* test with Bonferroni correction was applied.

## Results

### Effect of bilobalide on neurological deficit scores

**Figure 2:**
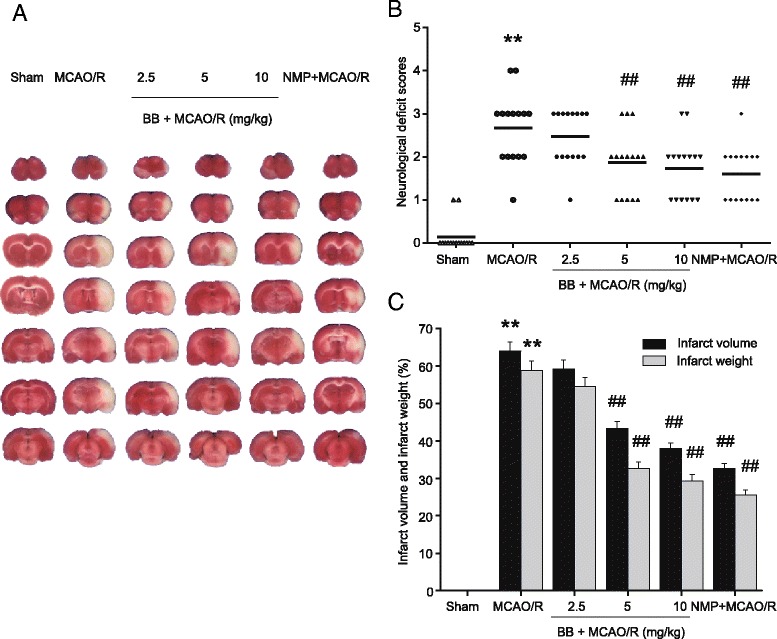
**Effects of bilobalide on neurological deficit scores, infarct volume, and infarct weight. (A)** Representative photographs of brain slices following infarction, stained with 2, 3, 5-triphenyltetrazolium chloride. Red tissue is healthy; white tissue is infarcted. Pretreatment with bilobalide (5, 10 mg/kg) significantly reduced infarct size **(B)** and improved neurological scores **(C)** and decreased infarct volume and infarct weight compared with the MCAO/R group. Mean values ± standard error of the mean for 15 (neurological evaluation) and six rats per group. ^**^ *P <* 0.01 versus sham; ^##^ *P <* 0.01 versus MCAO/R. BB, bilobalide; MCAO/R, middle cerebral artery occlusion and reperfusion; NMP, nimodipine.

After 2 h of ischemia followed by 24 h of reperfusion, rats subjected to MCAO showed significant motor behavioral deficits. Neurological deficit scores were significantly increased in the MCAO/R group (*P* < 0.01, Figure 2B). Administration of bilobalide (5, 10 mg/kg) and nimodipine resulted in a significant decrease in neurological scores in comparison with the MCAO/R group (*P* < 0.01, Figure 2B). There were no statistical differences in the scores between the bilobalide (2.5 mg/kg) and MCAO/R groups (*P* > 0.05, Figure 2B).

### Effects of bilobalide on infarct volume, infarct weight, and brain water content

Extensive infarction was detected by TTC staining in the cerebral cortical and subcortical areas over a series of sections of the ipsilateral hemisphere in rats subjected to MCAO (Figure 2A). Rats pretreated with both bilobalide (5, 10 mg/kg) and nimodipine had significantly smaller corrected infarct volumes than those in the MCAO/R group (*P* < 0.01, Figure 2A,C). Moreover, there was a significant increase in infarct weight after 24 h of reperfusion, whereas bilobalide (5, 10 mg/kg) and nimodipine pretreatment remarkably reduced infarct weight in brain sections (*P* < 0.01, Figure 2C).

**Figure 3:**
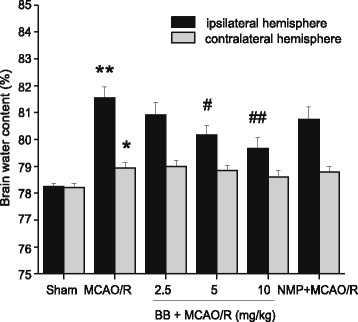
**Effect of bilobalide on brain water content.** Pretreatment with bilobalide (5, 10 mg/kg) significantly reduced brain water content in the ipsilateral hemisphere, but did not affect brain water content in the contralateral hemisphere compared with the MCAO/R group. Mean values ± standard error of the mean for six rats per group. ^*^ *P <* 0.05, ^**^ *P <* 0.01 versus sham; ^#^ *P <* 0.05, ^##^ *P <* 0.01 versus MCAO/R. BB, bilobalide; MCAO/R, middle cerebral artery occlusion and reperfusion; NMP, nimodipine.

Brain water content was examined to assess brain edema in both ipsilateral and contralateral hemispheres of all the groups. Brain water content was remarkably increased in the ipsilateral hemisphere in the MCAO/R group, which was significantly reduced by bilobalide (5, 10 mg/kg) pretreatment (*P* < 0.05, Figure 3). In contrast, MCAO induced a much slighter increase in brain water content in the contralateral hemisphere (Figure 3). In addition, pretreatment with bilobalide and nimodipine did not affect brain water content in the contralateral hemisphere in comparison with the MCAO/R group (*P* > 0.05, Figure 3). Bilobalide pretreatment at a lower dose (2.5 mg/kg) failed to limit cerebral infarct volume, infarct weight, and brain edema (*P* > 0.05, Figures 2A,C, 3). No infarction and edema formation were observed in the sham operation group (Figures 2A,C, 3).

### Effects of bilobalide on the levels of SOD and MDA

**Figure 4:**
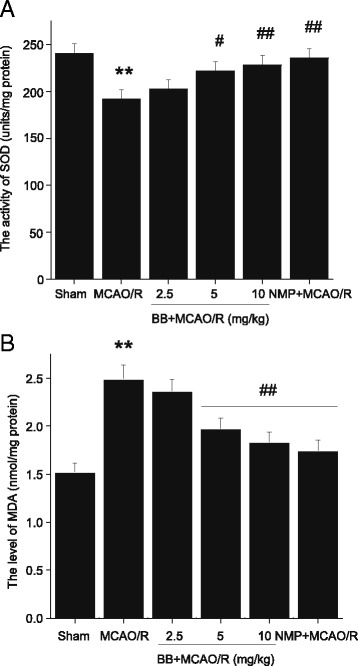
**Effects of bilobalide on the levels of SOD and MDA. (A)** Pretreatment with bilobalide (5, 10 mg/kg) significantly increased SOD activity **(B)** and decreased MDA concentration compared with the MCAO/R group. Mean values ± standard error of the mean for six rats per group. ^**^ *P <* 0.01 versus sham; ^#^ *P <* 0.05, ^##^ *P <* 0.01 versus MCAO/R. BB, bilobalide; MCAO/R, middle cerebral artery occlusion and reperfusion; NMP, nimodipine.

The activity of SOD was significantly lower and the concentration of MDA was higher in the ischemic penumbra of the cerebral cortex in the MCAO/R group than in the sham operation group (*P* < 0.01, Figure 4). Both bilobalide (5, 10 mg/kg) and nimodipine pretreatment significantly increased SOD activity and decreased MDA level, whereas bilobalide (2.5 mg/kg) did not change the levels of SOD and MDA significantly (Figure 4).

### Effects of bilobalide on the levels of nitric oxide, TNF-α, and IL-1β in ischemic penumbra after MCAO/R

**Figure 5:**
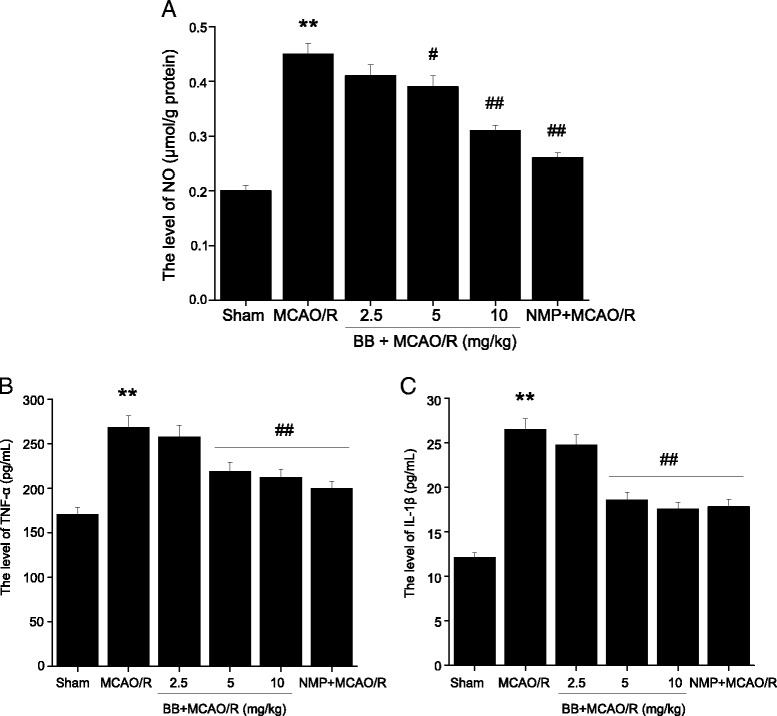
**Effects of bilobalide on the levels of nitric oxide, TNF-α, and IL-1β in ischemic penumbra after MCAO/R. (A)** Pretreatment with bilobalide (5, 10 mg/kg) significantly reduced concentrations of nitric oxide **(B)** and TNF-α **(C)** and IL-1β in the ischemic penumbra compared with the MCAO/R group. Mean values ± standard error of the mean for six rats per group. ^**^ *P <* 0.01 versus sham; ^#^ *P <* 0.05, ^##^ *P <* 0.01 versus MCAO/R. BB, bilobalide; MCAO/R, middle cerebral artery occlusion and reperfusion; NMP, nimodipine.

To explore whether bilobalide pretreatment could induce an anti-inflammatory pattern, we investigated the expression of three pro-inflammatory mediators in the ischemic penumbra of the cerebral cortex after 24 h of reperfusion by ELISA and specific assay kit. The MCAO/R group had significantly higher expression of nitric oxide, TNF-α, and IL-1β than did the sham group (*P* < 0.01, Figure 5). In comparison with the MCAO/R group, bilobalide (5, 10 mg/kg) and nimodipine pretreatment significantly reduced concentrations of nitric oxide, TNF-α, and IL-1β in the ischemic penumbra (*P* < 0.05, Figure 5). There were no significant differences between the bilobalide (2.5 mg/kg) and MCAO/R groups (*P >* 0.05, Figure 5).

### Effects of bilobalide on the expression of ERK1/2, JNK1/2, and p38 MAPK in ischemic penumbra after MCAO/R

**Figure 6:**
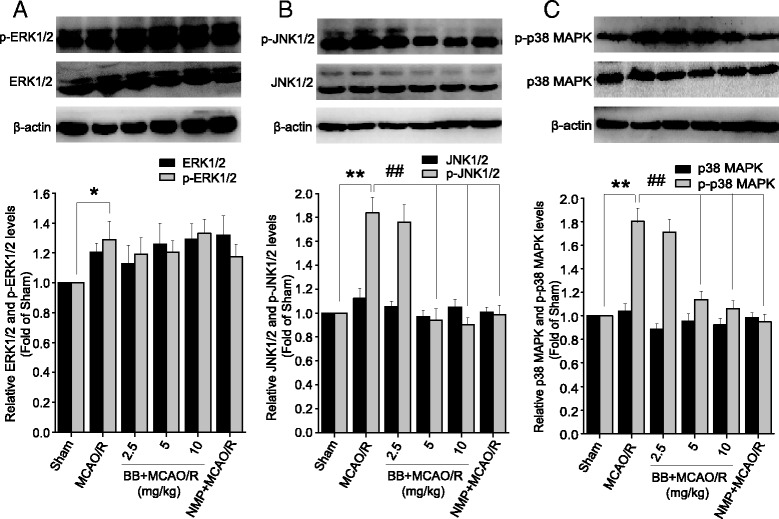
**Effects of bilobalide on the expression of ERK1/2, JNK1/2, and p38 MAPK in ischemic penumbra after MCAO/R. (A)** Pretreatment with bilobalide (5, 10 mg/kg) did not change p-ERK1/2 concentration, **(B)** significantly reduced p-JNK1/2 concentration, **(C)** and remarkably decreased p-p38 MAPK concentration in the ischemic penumbra compared with the MCAO/R group. There were no obvious differences in the levels of total ERK1/2, total JNK1/2, and total p38 MAPK among all experimental groups. Mean values ± standard error of the mean for six rats per group. ^*^ *P <* 0.05, ^**^ *P <* 0.01 versus sham; ^##^ *P <* 0.01 versus MCAO/R. BB, bilobalide; MCAO/R, middle cerebral artery occlusion and reperfusion; NMP, nimodipine.

Western blot analysis at 24 h following reperfusion showed, in the ischemic penumbra in MCAO/R rats, a significantly increased expression of the active p-ERK1/2, p-JNK1/2, and p-p38 MAPK in comparison with that of sham-operated animals (*P* < 0.05, Figure 6). In rats pretreated with bilobalide (5, 10 mg/kg) and nimodipine prior to MCAO there was a remarkable decrease in the levels of p-JNK1/2 and p-p38 MAPK, whereas p-ERK1/2 was unaffected, as compared with MCAO/R animals (Figure 6). Bilobalide (2.5 mg/kg) pretreatment did not change the levels of p-ERK1/2, p-JNK1/2, or p-p38 MAPK significantly (*P >* 0.05, Figure 6). There were no obvious differences in the levels of total ERK1/2, total JNK1/2, and total p38 MAPK among all experimental groups (*P >* 0.05, Figure 6).

### Effect of bilobalide on cell viability in primary cortical neurons after OGD/R

**Figure 7:**
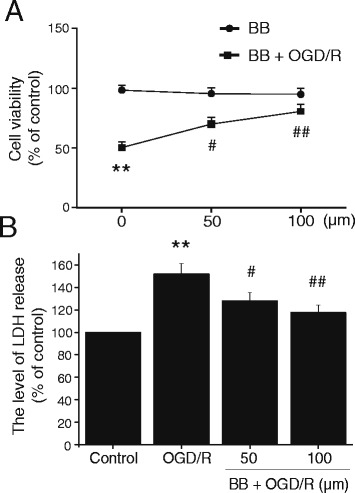
**Effects of bilobalide on cell viability in primary cortical neurons after OGD/R. (A)** Incubation of cortical neurons with different concentrations of bilobalide (50, 100 μM) alone for 12 h did not affect cell viability. After cortical neurons were exposed to 2 h of OGD following 24 h of reoxygenation, cell viability was significantly decreased; pretreatment with bilobalide (50, 100 μM) for 12 h significantly the cell viability. **(B)** After cortical neurons were exposed to 2 h of OGD following 24 h of reoxygenation, the level of LDH release was remarkably increased; pretreatment with bilobalide (50, 100 μM) for 12 h significantly decreased the LDH level. Mean values ± standard error of the mean ^**^ *P <* 0.01 versus control; ^#^ *P <* 0.05, ^##^ *P <* 0.01 versus*.* OGD/R. BB, bilobalide; LDH, lactate dehydrogenase; OGD/R, oxygen-glucose deprivation and reoxygenation.

Neuronal viability was assessed by both the MTT assay and measurement of extracellular LDH activity. After primary cortical neurons were exposed to 2 h of OGD following 24 h of reoxygenation, cell viability was significantly decreased and the level of LDH release was remarkably increased (*P* < 0.01, Figure 7). By contrast, incubation of cells with different concentrations of bilobalide (50, 100 μM) alone for 12 h did not affect cell viability (Figure 7A). Furthermore, pretreatment of cortical neurons with bilobalide (50, 100 μM) for 12 h, significantly increased the cell viability and decreased the LDH concentration (*P* < 0.05, Figure 7).

### Effects of bilobalide on the levels of nitric oxide, TNF-α, and IL-1β in primary cortical neurons after OGD/R

**Figure 8:**
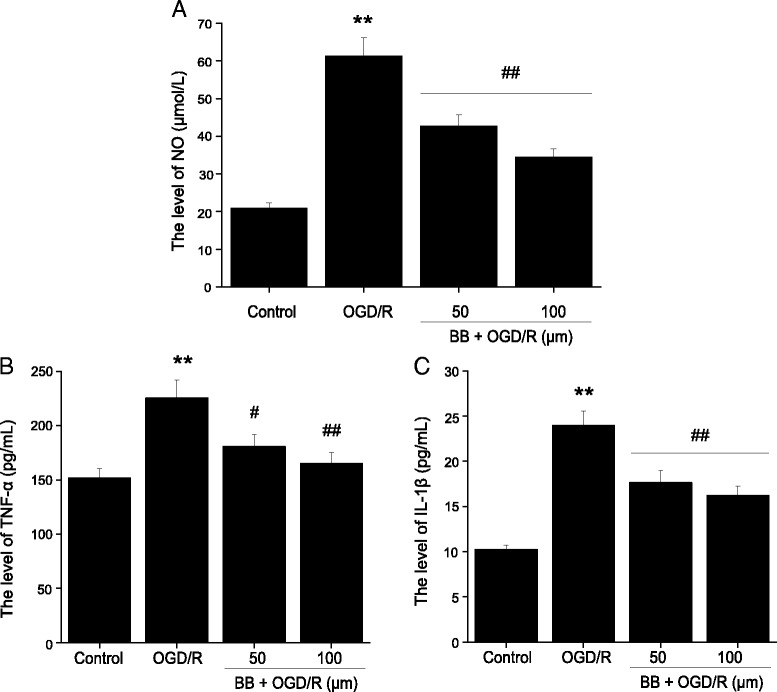
**Effects of bilobalide on the levels of nitric oxide, TNF-α, and IL-1β in primary cortical neurons after OGD/R. (A)** Pretreatment with bilobalide (50, 100 μM) significantly reduced nitric oxide **(B)** and TNF-α **(C)** and IL-1β levels in OGD/R-induced cortical neurons compared with the OGD/R group. Mean values ± standard error of the mean ^**^ *P <* 0.01 versus control; ^#^ *P <* 0.05, ^##^ *P <* 0.01 versus OGD/R. BB, bilobalide; OGD/R, oxygen-glucose deprivation and reoxygenation.

To examine further whether bilobalide pretreatment could associate with inhibition of pro-inflammatory mediator production, we measured the concentrations of nitric oxide, TNF-α, and IL-1β in OGD/R-induced cortical neurons. A similar pattern was observed in primary cortical neurons exposed to 2 h of OGD followed by 24 h of reoxygenation. In comparison with the control group, the expression of nitric oxide, TNF-α, and IL-1β significantly increased in OGD/R group (*P* < 0.01, Figure 8). As expected, pretreatment with bilobalide (50, 100 μM) led to a suppression on nitric oxide, TNF-α, and IL-1β expression in OGD/R-induced cortical neurons (*P* < 0.05, Figure 8).

### Effects of bilobalide on the expression of ERK1/2, JNK1/2, and p38 MAPK in primary cortical neurons after OGD/R

**Figure 9:**
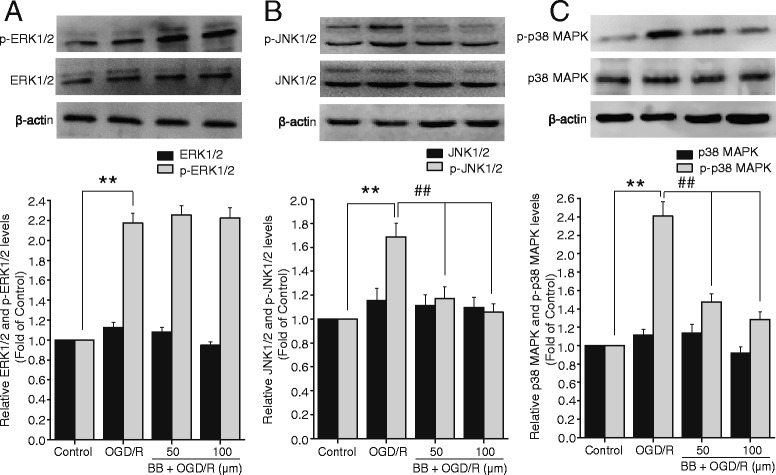
**Effects of bilobalide on the expression of ERK1/2, JNK1/2, and p38 MAPK in primary cortical neurons after OGD/R. (A)** Pretreatment with bilobalide (50, 100 μM) did not have an effect on p-ERK1/2 level **(B)** and significantly reduced p-JNK1/2 level **(C)** and remarkably down-regulated p-p38 MAPK expression in OGD/R-induced cortical neurons compared with the OGD/R group. No significant differences were observed in the expression of total ERK1/2, total JNK1/2, and total p38 MAPK among all experimental groups *in vitro*. Mean values ± standard error of the mean ^**^ *P <* 0.01 versus control; ^##^ *P <* 0.01 versus OGD/R. BB, bilobalide; OGD/R, oxygen-glucose deprivation and reoxygenation.

To further assess whether bilobalide pretreatment would modulate the MAPK pathways *in vitro*, we also examined the expression of p-ERK1/2, p-JNK1/2, and p-p38 MAPK in OGD/R-induced cortical neurons by Western blotting. Consistent with results *in vivo*, in primary cortical neurons exposed to 2 h of OGD followed by 24 h of reoxygenation, there was a significant up-regulation of p-ERK1/2, p-JNK1/2, and p-p38 MAPK in comparison with control neurons (*P* < 0.01, Figure 9). As expected, in OGD/R-induced cortical neurons bilobalide (50, 100 μM) pretreatment significantly down-regulated p-JNK1/2 and p-p38 MAPK, but did not have an effect on p-ERK1/2 expression (Figure 9). No significant differences were observed in the expression of total ERK1/2, total JNK1/2, and total p38 MAPK among all experimental groups *in vitro* (*P >* 0.05, Figure 9).

## Discussion

Since focal ischemia in the territory of the middle cerebral artery is the dominating cause of clinical stroke, the experimental model of MCAO is widely used in the study of ischemic stroke [60]. Focal cerebral ischemia is induced by temporary occlusion of the proximal portion of the right middle cerebral artery using the intraluminal suture method in rats. Reperfusion remains the treatment of choice for limiting brain injury following stroke, while restoration of blood flow is frequently associated with an exacerbation of tissue injury and a profound inflammatory response called reperfusion injury. Focal cerebral ischemia, particularly reperfusion injury, triggers multiple and distinct but overlapping cell signaling pathways in the brain, which may lead to cell survival or damage [61,62]. However, the mechanisms involving neuronal fate following I/R insult are complex and not fully understood. There is increasing evidence to show that MAPK signaling pathways become activated and play vital roles after focal cerebral I/R injury [7-10,13,14].

Bilobalide, a predominant sesquiterpene trilactone constituent of *Ginkgo biloba* leaves, has already been shown to exert potent neuroprotective and anti-apoptotic properties [31,32,45]. Since the first beneficial effect of bilobalide was detected on cytotoxic brain edema caused by triethyltin [63], several reports have not only revealed its diverse pharmacological properties but have also raised some speculative proposals concerning its mechanism of action [31-38]. Bilobalide has been demonstrated to have a protective effect on infarct volume in the rat model of focal cerebral ischemia, but to have no effect on the rats following global ischemia [48]. Bilobalide has been proposed to exert protective and trophic effects on neurons, and act on astrocytes that contribute strongly to brain swelling [31,39]. In addition, attenuation of neuronal damage and apoptosis by bilobalide was closely related to suppression of the up-regulation of c-*myc*, p53, *bax*, TNF-α, Aβ_1–40_ expression, and activation of caspase-3 [31,42]. Recent evidence has further shown that the protective effects of bilobalide against neuronal apoptosis were associated with increasing PI3K activity and up-regulation of phosphorylated Akt in a dose-dependent manner, and could be abrogated by the PI3K/Akt inhibitor LY294002 [45]. Taken together, these studies clearly show that bilobalide protects against cerebral I/R injury and suggest that modulation of both anti-apoptotic signaling cascades and pro-inflammatory mediators might underlie this protection.

In this study, we used MCAO/R and OGD/R models of cerebral I/R injury to further investigate the neuroprotective effects of bilobalide as well as the underlying mechanisms, by focusing on MAPK signaling pathways, as these pathways were closely related to inflammatory response and apoptotic signaling. In line with previous studies, our results indicated that bilobalide had significant neuroprotective effects against cerebral I/R injury. The evidence was that bilobalide improved neurological function, decreased infarct volume, and ameliorated brain edema when administered intraperitoneally at doses of 5 and 10 mg/kg 60 min prior to MCAO. Previous studies have shown that when administered either 60 min before MCAO or immediately after ischemia, bilobalide reduced infarct volume significantly, whereas no effect was demonstrated when bilobalide was administered 60 min after ischemia [48]. Our results indicated clearly that bilobalide, when administered 60 min before MCAO, significantly reduced infarct volume and infarct weight. A strong beneficial effect of bilobalide on anti-edema formation has been detected in models of brain ischemia *in vivo* and *in vitro* [39]. Thus, bilobalide may be developed as a potential anti-edema drug. As reported by previous studies, brain edema reached its peak 24 h following ischemia in experimental models [64,65]. Here we showed that bilobalide pretreatment decreased brain water content significantly, whereas nimodipine did not. Interestingly, our data demonstrated that the brain water content was significantly increased in both the ipsilateral and contralateral hemispheres in the MCAO/R group, although infarct areas by TTC staining were just detected in the ipsilateral hemisphere. Furthermore, bilobalide had an anti-edemic effect in the ipsilateral hemisphere but did not have any effect on the contralateral hemisphere. Bilobalide’s anti-edema effect was involved in improving the maintenance of ionic balances, and astrocytes might be the target [66,67]. Since an outstanding anti-inflammatory effect of bilobalide has been reported recently [33], we assume that the anti-edema mechanisms of bilobalide might also relate to its anti-inflammatory activity.

It has been well proven that cerebral ischemia significantly increases the content of reactive oxygen species, such as hydroxyl radical, superoxide anion, and hydrogen peroxide, and decreases the activity of antioxidant enzymes in the cerebral cortex [68,69]. In this study, we showed that bilobalide pretreatment significantly increased SOD activity and decreased MDA levels in the ischemic penumbra of the cerebral cortex. Thus, our results have provided evidence for bilodalide’s antioxidative activity after focal cerebral I/R.

Focal cerebral I/R injury causes a robust increase in typical markers of inflammation, such as nitric oxide, TNF-α, and IL-1β, which are involved in the process of cell damage. Recent studies reported that many types of actions induced by TNF-α and IL-1β were mediated by caspase pathways and inducible nitric oxide synthase-derived nitric oxide [70]. It has been suggested that nitric oxide is detrimental in ischemic brain injury whereas evidence also supports that nitric oxide produced by inducible nitric oxide synthase may act as a neurotrophic agent by promoting the differentiation and survival of neurons [71]. Taken together, nitric oxide, TNF-α, and IL-1β have important roles in the pathogenesis of cerebral I/R injury. Here, we showed that the expression of nitric oxide, TNF-α, and IL-1β in MCAO/R rats were significantly higher than those in sham animals. According to more recent findings, bilobalide has an excellent anti-inflammatory effect [33] and could inhibit TNF-α expression in the frontal cortex and hippocampus CA1 of Alzheimer’s disease model rats [42]. Similarly, we showed that pretreatment with bilobalide significantly reduced the expression of nitric oxide, TNF-α, and IL-1β in the ischemic penumbra.

Neuronal damage after I/R injury occurs via oxidative stress, inflammation response, or mitochondrial dysfunction, and ultimately activates an apoptotic cascade. These events demonstrate overlapping and redundant features and depend on the intensity and duration of ischemic insult. The importance of MAPK signaling pathways as both targets and mediators of cerebral I/R injury is becoming increasingly recognized. Oxidative stress and inflammatory mediators produced after the onset of cerebral I/R have been shown to activate MAPK signaling cascades that participate in neuronal survival or damage [8,10,13]. Our results demonstrated that phosphorylation of ERK1/2, JNK1/2, and p38 MAPK protein were significantly increased in the ischemic penumbra in MCAO/R rats. Bilobalide inhibited JNK1/2 and p38 MAPK activation, but had no effect on the increased p-ERK1/2 expression. Furthermore, we examined the neuroprotective effects of bilobalide on OGD/R-induced cortical neurons. The results showed that pretreatment of cortical neurons with bilobalide significantly increased the cell viability and decreased the LDH concentration. Consistent with results *in vivo*, there was also a significant up-regulation of p-ERK1/2, p-JNK1/2, and p-p38 MAPK in primary cortical neurons exposed to 2 h of OGD followed by 24 h of reoxygenation. Interestingly, the levels of p-JNK1/2 and p-p38 MAPK were also significantly down-regulated in bilobalide-treated cells, and no change in the expression of p-ERK1/2 was observed. These results suggested that JNK1/2 and p38 MAPK pathways could be involved in the neuroprotective effects of bilobalide. It is known that, the activated JNK and p38 MAPK mainly function as mediators of cellular stress in cerebral I/R injury by phosphorylating intracellular enzymes, transcription factors, and cytosolic proteins involved in cell survival, inflammatory mediators production, and apoptosis [8]. Phosphorylated ERK, JNK, and p38 MAPK activated their phosphorylated substrates such as CREB, c-*Myc*, Elk-1, ATF-2, and c-Jun, and increased expression of p-CREB, p-Elk-1, p-c-*Myc*, p-ATF-2, and p-c-Jun occurred in the infarct penumbra at 4 h following reperfusion [9]. Active JNK has been shown to stabilize wild-type p53 by phosphorylation similar to c-Jun and JNK-mediated phosphorylation increases p53-dependent *trans*-activation and potentiates p53-mediated cell death. In cerebral ischemia, increased neuronal expression of p53 and activation of *bax*, a pro-apoptotic p53 target gene, have been reported [72]. Moreover, the PI3K-Akt pathway prevented neuronal apoptosis by suppressing the activation of JNK and c-Jun expression [13]. Interestingly, a recent study showed that activation of the PI3K/Akt pathway by bilobalide blocked cell apoptosis in SH-SY5Y cells [45]. These studies indicated that the protective effects of bilobalide against neuronal apoptosis could associate with suppressing the activation of JNK1/2. Phosphorylated p38 MAPK can phosphorylate MAP kinase AP-2 and MAP kinase AP-3, which regulate Hsp-27, increase the expression of pro-inflammatory cytokines such as TNF-α and IL-6, induce an inflammatory cascade, and increase cellular damage [73,74]. Suppression of p38 MAPK phosphorylation probably reduces neuronal cell death by inhibiting the production of inflammatory mediators [18]. In many cell death models, p38 MAPK acts upstream of caspase execution. Therefore, regulation of neuronal damage by JNK and p38 MAPK pathways is related to multiple downstream transcription factors and apoptosis-associated proteins. More interestingly, previous studies showed that bilobalide shared common downstream targets with the JNK and p38 MAPK pathways such as CREB, c-*myc*, p53, *bax*, caspase-3, TNF-α, and even the PI3K-Akt pathway [31,42,45]. These findings, together with our results, support the involvement of the JNK1/2 and p38 MAPK pathways in the neuroprotective effects of bilobalide. Notably, bilobalide failed to inhibit the increased p-ERK1/2 expression; this was consistent with previous observations that bilobalide treatment did not change p-ERK1/2 levels in SH-SY5Y cells [45].

Our data indicated that there was also a significant up-regulation of nitric oxide, TNF-α, IL-1β, p-ERK1/2, p-JNK1/2, and p-p38 MAPK in primary cortical neurons exposed to 2 h of OGD followed by 24 h of reoxygenation. This is consistent with previous reports regarding the production of pro-inflammatory mediators [75-77] and the activation of MAPK pathways [8,78] in cultured cortical neurons after OGD/R. However, pretreatment with bilobalide significantly down-regulated nitric oxide, TNF-α, IL-1β, p-JNK1/2, and p-p38 MAPK concentration in primary cortical neurons after OGD/R. Bilobalide may also affect inflammatory and apoptotic processes in non-neuronal cell types (for example, microglia, astrocytes, endothelial cells) which were spared in ischemic brain. Further work is necessary to clarify this point. Nevertheless, the results generated by using an *in vitro* OGD/R model convincingly demonstrate a prominent role for bilobalide in cerebral I/R injury.

Previous evidence has suggested that bilobalide crosses the blood-brain barrier easily and rapidly (and bilobalide can remain in brain tissue in a prolonged manner after ischemia has been induced), and reaches extracellular concentrations in brain that allow efficient interaction with target molecules, such as neurotransmitter receptors [79]. Moreover, bilobalide, when given before MCAO, remains in the brain tissue for extended periods of time even after disruption of blood flow [79]. Therefore, the availability of bilobalide in brain is evidently sufficient to explain the neuroprotective effects of bilobalide. The clinical studies, in addition, showed that bilobalide was highly bioavailable, with bioavailability of 70%, and well tolerated [47,80]. In particular, bilobalide can be given in a preventive manner with little potential for adverse side effects.

## Conclusions

In summary, this study demonstrated that the neuroprotective effects of bilobalide on cerebral I/R injury were associated with attenuation of nitric oxide, TNF-α, and IL-1β production and suppression of JNK1/2 and p38 MAPK activation. Although further studies are needed to elucidate the roles of MAPK signaling pathways in the cross talk between pro-inflammatory mediators and apoptosis, our findings may represent a novel mechanism of bilobalide in focal cerebral I/R injury in rats (Figure 1B). Since bilobalide has originally been identified as a potential neuroprotective drug, our results may provide new insight into therapeutic targets of bilobalide in patients with neurodegenerative disorders, such as stroke, Alzheimer’s disease, and dementia by unknown causes.

## Abbreviations

ANOVA: analysis of variance
BSS: glucose-free Earle’s balanced salt solution
DMEM: Dulbecco’s modified Eagle’s medium
DMSO: dimethyl sulfoxide
ELISA: enzyme-linked immunosorbent assay
ERK: extracellular signal-regulated kinase
FCS: fetal calf serum
IL-1β: interleukin 1β
I/R: ischemia and reperfusion
JNK: c-Jun NH_2_-terminal kinase
LDH: lactate dehydrogenase
MAP: mitogen-activated protein
MAPK: mitogen-activated protein kinase
MCAO: middle cerebral artery occlusion
MCAO/R: middle cerebral artery occlusion and reperfusion
MDA: malondialdehyde
MTT: 3-(4,5-dimethylthiazol-2-yl)-2,5-diphenyl tetrazolium bromide
OGD/R: oxygen-glucose deprivation and reoxygenation
PBS: phosphate buffered saline
SOD: superoxide dismutase
TBST: Tris-buffered saline containing 0.05% Tween-20
TNF-α: tumor necrosis factor α
TTC: 2,3,5-triphenyltetrazolium chloride
